# Mpox in nonendemic regions: Case series of 2 Nepalese men residing in Saudi Arabia

**DOI:** 10.1016/j.jdcr.2025.08.029

**Published:** 2025-09-26

**Authors:** Priya Karna, Shivendra Jha

**Affiliations:** a Department of Dermatology, Sukraraj Tropical and Infectious Disease Hospital, Teku, Kathmandu; b Dermatology, Sukraraj Tropical and Infectious Disease Hospital, Teku, Kathmandu

**Keywords:** case series, infectious disease, mpox, Nepal, sexually transmitted disease (STDs)

## Introduction

Mpox virus (formerly known as monkeypox) is a zoonotic Orthopoxvirus DNA virus closely related to the smallpox virus. It was first identified in humans in the Democratic Republic of the Congo in 1970.^1^ The 2022 outbreak revealed a higher transmission rate through sexual networks, disproportionately affecting gay, bisexual, and other men who have sex with men (MSM).^2^^,^^3^ Vaginal and oral/perioral lesions, suggesting potential sexual transmission, were common presentations in the outbreak. As of August 27, 2022, 7 cases had been documented in Saudi Arabia.^4^ The resurgence of poxviruses is thought to be linked to the discontinuation of smallpox vaccination over 40 years ago, leading to diminished cross-immunity.^5^

Here, we present the history and clinical findings of 2 Nepalese men residing in Saudi Arabia with probable mpox infection.

Our report adds value by documenting 2 cases of mpox among migrant laborers in Saudi Arabia, highlighting clinical findings and health care challenges in an underrepresented and resource-limited population. These cases contribute to the broader understanding of mpox’s presentation and transmission among heterosexual individuals in nonendemic Gulf settings.

## Case report

### Case 1

A 36-year-old male, a permanent resident of Tanahun, Myagdi-4, Nepal, working as a plumber in Saudi Arabia for the past 10-11 years, presented with a 1-month history of papular and pustular skin rashes. He had visited Nepal 1 year prior. The patient reported sexual exposure to 3 high-risk females (sex workers) over 3-4 days, with condom use, except for 1 instance of condom breakage. The encounters were described as heterosexual.

Approximately 3-4 days after the exposure, he experienced mild fever, fatigue, lethargy, sore throat, and myalgia, without back pain, arthralgia, headache, or lymphadenopathy. Two days later, furuncle-like lesions developed at the lateral base of the penis, which progressed to pustular lesions, ruptured, and crusted. The rash followed a sequential pattern of appearance: right thumb and index finger web space → left forearm and arm → chest → upper back → scrotum → penis → face → soles → intergluteal region.

He denied penile swelling, discharge, rectal bleeding, tenesmus, dysuria, ocular symptoms, respiratory symptoms, gastrointestinal complaints, or weight loss. He reported no history of chronic illnesses and no prior smallpox vaccination.

On examination, over 50 well-defined papular and pustular lesions with crusting on an erythematous base were distributed asymmetrically across multiple body sites. Lesions ranged from 0.1 cm to 1 cm, were hemispherical, were deep-seated, and had smooth surfaces. No mucosal involvement or palpable lymphadenopathy was noted. Systemic examination was unremarkable.

A provisional diagnosis of probable mpox was made. Laboratory investigations, including complete blood count, complete metabolic panel, urine analysis, and serologies for HIV, hepatitis B, and syphilis, were within normal limits or negative. Samples were collected for mpox polymerase chain reaction (PCR) testing from lesions of various stages and a crust sample from the pubic region. PCR testing confirmed mpox infection.

Management included isolation, supportive care with hydration, a high-protein diet, maintenance of room temperature, and topical treatments with coconut oil and topical antibiotics. Oral medications included cefixime, azithromycin, cetirizine, and multivitamins.

**Fig 1:**
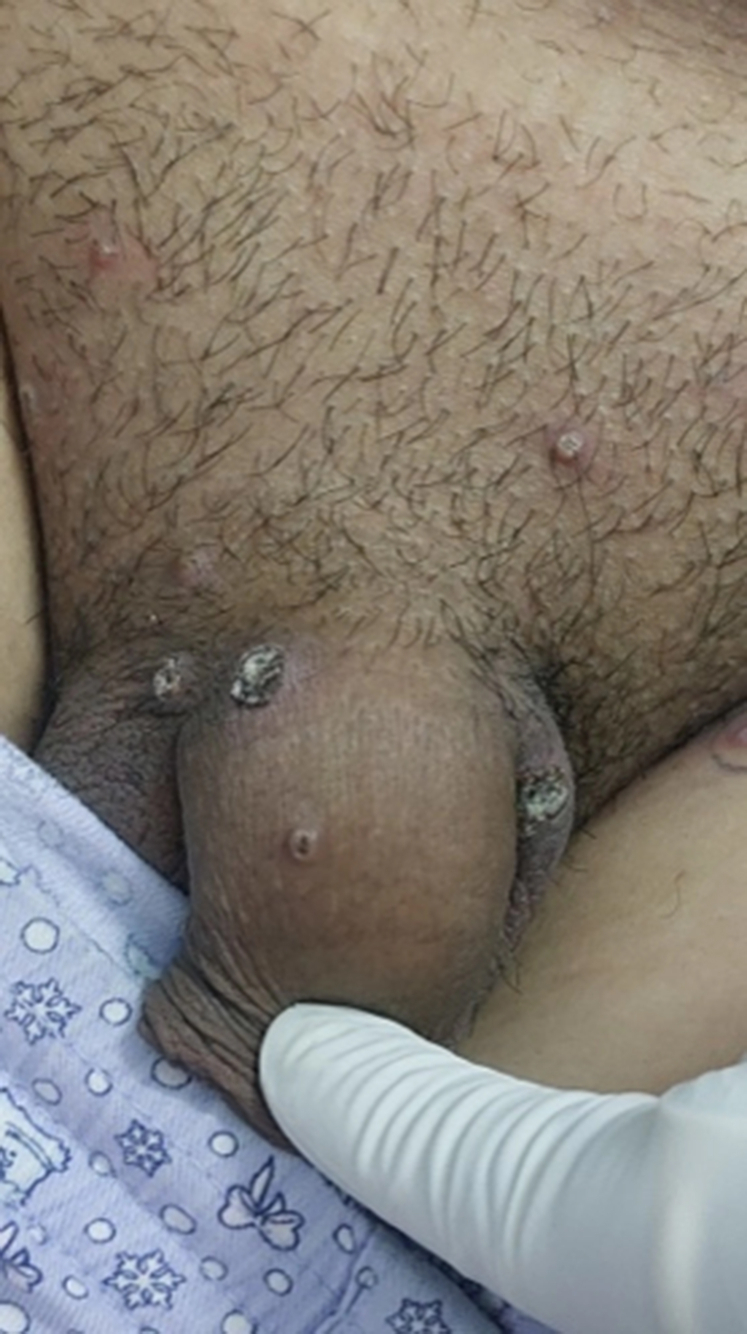
Multiple well-defined papular and pustular lesions with crusting distributed over genitalia at presentation in case 1.

**Fig 2:**
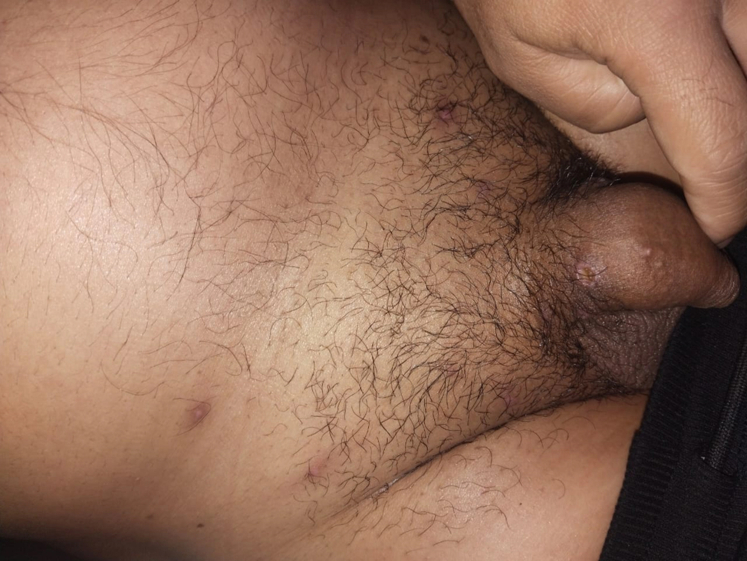
Significant healing and resolution of genital lesions in case 1 after 3 weeks of supportive care.

Image of lesions present over genital area has been shown in Figs 1 and 2 below.

### Case 2

A 44-year-old male, a permanent resident of Sindhuli, Marin-7, Nepal, working as a driver in Saudi Arabia for the past 16-17 years, presented with a 2-week history of a papular and pustular skin rash. He had last visited Nepal 1 year prior.

Seventeen days before the presentation, he developed an acute-onset continuous fever with severe chills and a headache that limited daily activities, partially relieved by medication. The fever improved slightly with NSAIDs. He also reported sore throat, myalgia, fatigue, and painful bilateral inguinal swelling (2 pea-sized nodes with mild erythema).

Two to 3 days later, a papular erythematous lesion appeared on the lateral aspect of the right leg, progressing to vesicular and pustular stages before rupturing and crusting over 5-6 days. New lesions appeared at a rate of 2-3 per day in the following sequence: right lateral leg → genital region → right palm → face → arms → chest → back → left shin (most recent, 3 days prior).

The patient denied itching, rectal bleeding, tenesmus, dysuria, ocular symptoms, respiratory symptoms, gastrointestinal symptoms, weight loss, or a history of sexual exposure. He had no chronic illnesses and no history of smallpox vaccination.

On examination, more than 40 well-defined papular and pustular lesions, some crusted, were asymmetrically distributed across the body. Lesions ranged from 0.1 cm to 1 cm, were hemispherical, deep-seated, and smooth-surfaced over an erythematous base. No oral or ocular involvement was noted. Genital examination showed severe swelling with inability to retract the prepuce, superficial punctate ulcers on the glans, and crusting obscuring the coronal sulcus. No palpable cervical or inguinal lymphadenopathy was present. Systemic examination was otherwise normal.

A provisional diagnosis of probable mpox was considered. Investigations, including complete blood count, complete metabolic panel, urine analysis, and serologies for HIV, hepatitis B, and syphilis, were normal or negative. Samples collected from lesions and crusts tested positive for mpox by PCR.

Management involved patient isolation, encouragement of oral intake, a high-protein diet, room temperature maintenance, topical wound care with coconut oil and antibiotics, and oral medications including cefixime, azithromycin, cetirizine, multivitamins, and prednisolone 30 mg once daily for 5 days.

## Discussion

Recent outbreaks of mpox in nonendemic regions have brought renewed attention to its transmission dynamics, clinical presentation, and diagnostic strategies. The cases presented here highlight these features and align with findings from other outbreaks.

The first patient reported high-risk sexual exposure prior to developing characteristic papular and pustular skin lesions that later crusted. This clinical course is consistent with findings by Adler et al.^6^ Although lymphadenopathy is a hallmark prodromal symptom, its absence, particularly in mild cases, is documented in the literature (McCollum and Damon).^7^

The geographic context is also significant. Saudi Arabia, a nonendemic region, reported 7 confirmed cases by August 2022,^8^ supporting the global spread described by Bunge et al.^9^ International travel and close contact among high-risk groups have been implicated in this spread.

Emerging research suggests sexual transmission, particularly among MSM, as a significant route. Thornhill et al found over 90% of cases in MSM, with lesions commonly affecting anogenital regions, supporting sexual transmission pathways.^2^ Although our patients reported heterosexual orientation (and in 1 case denied recent sexual exposure), the lesion distribution suggests the possibility of sexual transmission even in heterosexual contacts.

PCR testing from lesion samples remains the gold standard for diagnosis, as per World Health Organization recommendations.^10^ Comprehensive testing ruled out alternative infectious causes, including syphilis, herpes simplex virus, and varicella zoster, consistent with best clinical practices.

Supportive management, emphasizing isolation, hydration, and wound care, proved effective, as documented in previous studies.^11^ The lack of access to antiviral agents like tecovirimat, highlighted by Reynolds et al,^12^ reinforces the importance of early symptomatic management.

These cases underscore the challenges of Mpox diagnosis in resource-limited environments. While PCR testing was successfully performed here, access to such molecular diagnostics is rare and often contingent on occupational networks. In many similar settings, diagnosis depends on clinical judgment, lesion morphology, and syndromic protocols, as dermoscopy and point-of-care tests are generally unavailable.

Similarly, therapeutic limitations are evident. While World Health Organization recommends supportive care, the unavailability of antiviral therapies such as tecovirimat in low-income regions presents a clear equity gap. Our cases were managed with hydration, antibiotics, and topical care—reflecting common frontline strategies in underresourced areas. The outcomes highlight both the sufficiency and limitations of supportive therapy alone.

The migrant labor population in Gulf countries, often living in densely populated and medically underserved environments, is particularly vulnerable. Social stigma, poor health education, and systemic underreporting hinder timely diagnosis and care. Our patients’ access to testing and treatment through employer-linked health care systems may not be representative of broader migrant experiences.

Although genotyping was not available in our setting, current literature highlights the need to distinguish among clades. Since 2022, the global outbreak has been largely associated with clade IIb, while recent resurgences in Sierra Leone involve the more virulent clade IB.^13^ Our patients likely represent clade IIb infections given their geographic location and moderate clinical course. Continued investment in genomic surveillance is crucial for outbreak monitoring.

The resurgence of mpox underscores vulnerabilities related to waning cross-immunity following the cessation of smallpox vaccination, a trend noted in epidemiological studies such as Xiang et al.^13^ Our patients’ lack of smallpox vaccination further supports this theory.

The resurgence of mpox as a global health threat, particularly in nonendemic regions, highlights the disease’s evolving epidemiology. These 2 cases of probable mpox in Nepalese men residing in Saudi Arabia underscore the importance of early recognition of clinical manifestations, including papular and pustular skin lesions, systemic symptoms, and the potential for sexual transmission.

Timely diagnosis through PCR, early isolation, supportive management, and prevention of secondary infections proved effective. The absence of smallpox vaccination in both patients points to decreased cross-protective immunity as a contributing factor to susceptibility.

In summary, these cases highlight key diagnostic, therapeutic, and surveillance challenges in resource-limited nonendemic settings. Addressing disparities in health care access, antiviral availability, and public health outreach is vital in preparing for future outbreaks.

Continued surveillance, early diagnosis, public health interventions, and broader access to antivirals will be critical in controlling future outbreaks.

## Conflicts of interest

None disclosed.
